# Adapting the traditional case report to a biopsychosocial format

**DOI:** 10.1192/j.eurpsy.2021.1582

**Published:** 2021-08-13

**Authors:** R. Wynn, L. Myklebust

**Affiliations:** 1 Department Of Clinical Medicine, UiT The Arctic University of Norway, Tromsø, Norway; 2 Psychiatric Research Centre Of Northern Norway, Nordland Hospital Trust, Bodø, Norway

**Keywords:** medical literature, case report, biopsychosocial

## Abstract

**Introduction:**

The medical case report (CR) is a vital and viable medical genre with a history of more than 3000 years. With a few exceptions, the CR has had a typical format that has been consistent with the ideals of brevity, conciseness, and a matter-of-fact approach. CR in general and psychiatric CR especially, may benefit from more systematically emphasising and integrating relevant biopsychosocial (BPS) aspects.

**Objectives:**

To discuss how to emphasise and integrate the BPS perspective in the CR.

**Methods:**

Drawing on CR literature and our own experience as CR authors, we discuss how a broader BPS approach successfully can be included in the CR format.

**Results:**

Some central factors that could be considered when including a BPS perspective in the CR are: 1) Actively eliciting the patient’s perspective and including this in the final report. 2) Including relevant information about the life and circumstances of the patient beyond the basic demographic information. 3) Making an effort to preserve the patient’s privacy also when more BPS information is included. The psychological and social constituents of the patient’s life should be central in the BPS-inspired psychiatric CR.

**Conclusions:**

The traditional CR has a long-standing history in medicine and follows a typial conscise and brief format. ‘Hard facts’ and biological information have typically filled most of the text. We argue that giving psychological and social information more attention would improve the quality of many CR, and that this is especially relevant for psychiatric CR.

